# From nanoparticles to crystals: one-pot programmable biosynthesis of photothermal gold structures and their use for biomedical applications

**DOI:** 10.1186/s12951-022-01680-7

**Published:** 2022-11-16

**Authors:** Roman Nudelman, Hashim Alhmoud, Bahman Delalat, Ishdeep Kaur, Anastasia Vitkin, Laure Bourgeois, Ilan Goldfarb, Anna Cifuentes-Rius, Nicolas H. Voelcker, Shachar Richter

**Affiliations:** 1 Department of Material Science and Engineering, Center for Nano-Science and Nano-Technology, Tel-Aviv University, 69978 Tel-Aviv, Israel; 2 Monash Institute of Pharmaceutical Sciences, Monash University, Parkville Campus, 381 Royal Parade, Parkville, VIC 3052 Australia; 3 CSIRO Manufacturing, Bayview Avenue, Clayton, VIC 3168 Australia; 4 Monash Centre for Electron Microscopy, Department of Materials Science and Engineering, Faculty of Engineering, Monash University, Clayton Campus, 10 Innovation Walk, Clayton, VIC 3168 Australia; 5 Melbourne Centre for Nanofabrication, Victorian Node of the Australian National Fabrication Facility, 151 Wellington Road, Clayton, VIC 3168 Australia

**Keywords:** Protein-templated synthesis, Gold nanoparticles, Mucin, Green synthesis, Photothermal Materials, Antibacterial activity

## Abstract

Inspired by nature, green chemistry uses various biomolecules, such as proteins, as reducing agents to synthesize metallic nanostructures. This methodology provides an alternative route to conventional harsh synthetic processes, which include polluting chemicals. Tuning the resulting nanostructure properties, such as their size and shape, is challenging as the exact mechanism involved in their formation is still not well understood. This work reports a well-controlled method to program gold nanostructures' shape, size, and aggregation state using only one protein type, mucin, as a reduction and capping material in a one-pot bio-assisted reaction. Using mucin as a gold reduction template while varying its tertiary structure via the pH of the synthesis, we demonstrate that spherical, coral-shaped, and hexagonal gold crystals can be obtained and that the size can be tuned over three orders of magnitude. This is achieved by leveraging the protein's intrinsic reducing properties and pH-induced conformational changes. The systematic study of the reaction kinetics and growth steps developed here provides an understanding of the mechanism behind this phenomenon. We further show that the prepared gold nanostructures exhibit tunable photothermal properties that can be optimized for various hyperthermia-induced antibacterial applications.

**Supplementary Information:**

The online version contains supplementary material available at 10.1186/s12951-022-01680-7.

## Background

Nature produces complex materials at the nanoscale with exquisite precision and reproducibility [1–3]. In this respect, extracts of plants, bacteria, fungi, and specialized proteins [4–8] can induce, template, or catalyze the synthesis of nanomaterials such as metallic nanostructures [9, 10].

One of the environmentally benign ways for the green and bio-templated synthesis of metallic nanostructures is using proteins that exhibit strong chemical reduction capabilities [5, 11, 12]. These usually consist of amino acids such as tyrosine, tryptophan, aspartic acid, and cysteine (Cys) [13, 14] that can attract positive metal ions and conduct electron transfer and chemically reduce the metal ions in their vicinity.

Although many metal nanostructures have been reported via such bio-templated approaches [15–18], full and simultaneous control over the nanostructure size, shape, structure, and aggregation arrangements while using the same protein remains a grand challenge. This is most likely due to the complexity of predicting, controlling, and tuning the tertiary conformation of the protein and the number of its available chemical reduction sites [19–21]. Moreover, due to the complexity of the process, understanding the mechanism of the reactions involved and the nucleation and growth steps is very challenging.

It has been previously suggested [19, 21] that control over the protein's tertiary structure is essential for obtaining the desired products. This can be achieved by tuning the pH of the reaction, as demonstrated in of mucins proteins [22, 23], and in particular in the case of porcine gastric mucin (PGM) [24–27] a high molecular weight type of glycoprotein [28–31].

In this case, tuning of the synthesis is governed by the reduction sites available at certain pH, the ternary structure of the protein, and the intermolecular bonds between the mucins: The PGM's skeleton comprises many repeated sequences of amino acids, including Cys, present mainly in the protein core's hydrophobic pockets [32, 33]. In particular, Cys’ thiol side-group possesses unique chemical characteristics such as nucleophilicity, redox activity, and metal-binding properties for nanostructure synthesis [34, 35]. The Cys’ disulfide bonds are exposed to the surroundings under acidic conditions due to the breakage of salt bridges between negatively charged carboxylates and positively charged amino groups [36, 37]. This unfolded form facilitates the formation of new disulfide intermolecular bonds between neighboring proteins [38–40]. At this pH and low PGM concentrations, intramolecular interactions between the hydrophobic domains occur in the same protein unit. Increasing the PGM concentration causes intramolecular interactions between adjacent protein units, thus transforming the protein solution into a sol–gel form [41, 42]. This property is responsible for the pH-dependent viscoelastic properties of mucin-based biomasses in nature [43, 44]. In contrast, the PGM pockets remain closed or partially folded under alkaline or neutral conditions, and mainly weak intermolecular interactions between its monomers occur [43–45]. In this state, the PGM is dispersed, and aggregation is substantially reduced due to hydrophobic interactions [46, 47]. Thus, in this range, the available chemical reduction sites for the synthesis are less exposed than in acidic conditions [45, 47–49].

Based on these observations, we hypothesized that the pH environment defines the hydrophobic pocket's volume in which the electron transfer between the protein and metal cations occurs and also the number of available reduction sites needed for the synthesis [50, 51]: in alkaline conditions, the synthesis takes place in an isolated small volume, while in an acidic environment, the opposite is true, and synthesis occurs in large volumes [52, 53]. Also, it can be assumed that the PGM concentration affects the reaction rate of metal nanostructures formation due to the gelation process, which slows down the nucleation kinetics and increases the reaction time until the formation of final nanostructures.

When Gold (Au) nanoparticles (AuNP) are synthesized in this manner, they can be used as photothermal (PT) agents [54, 55]. When irradiated, the energy is transformed into heat and can be used for various applications, including hyperthermia treatments [55, 56]. The major mechanism of this phenomenon is the PT due to absorbance at the plasmonic resonance, which can be tuned by means of the size of the AuNP[57]. However, it was shown that even the off-resonance PT effect could be present in such a system which may be originated from light localization effects [57, 58].

Recently, AuNp have been investigated as a possible treatment agent for various tumors, especially melanoma skin cancer [56]. This was possible due to the biocompatibility of AuNp and versatile chemistry, which can be performed to attach different organic molecules to the AuNp surface to turn them into drug carrier particles. Importantly, these could also be used in localized PT treatment in which AuNp accumulates in the vicinity of cancerous growth due to enhanced permeability and retention effect. This allows the application of the PT effect in order to inhibit tumors or even destroy them. Moreover, photothermal AuNp has recently come under the spotlight as an alternative therapy to treat stubborn infections [59], especially with the rise of various antibiotic-resistant strains of pathogenic bacteria where conventional antibiotics are no longer effective. This becomes especially important in chronic wounds infected with methicillin-resistant *S. aureus* (MRSA) and Vancomycin-resistant *Enterococci*. Near-infrared (NIR) radiation suffers very little scattering in biological tissue and is virtually harmless (unlike UV and other high-energy radiation). Therefore, it is ideal for delivering hyperthermal energy to infected wounds [55]. AuNP can act as a localized nanoscale heat source for converting the NIR radiation into heat, potentially disrupting bacterial membranes and leading to the denaturation of bacteria.

**Scheme 1.**
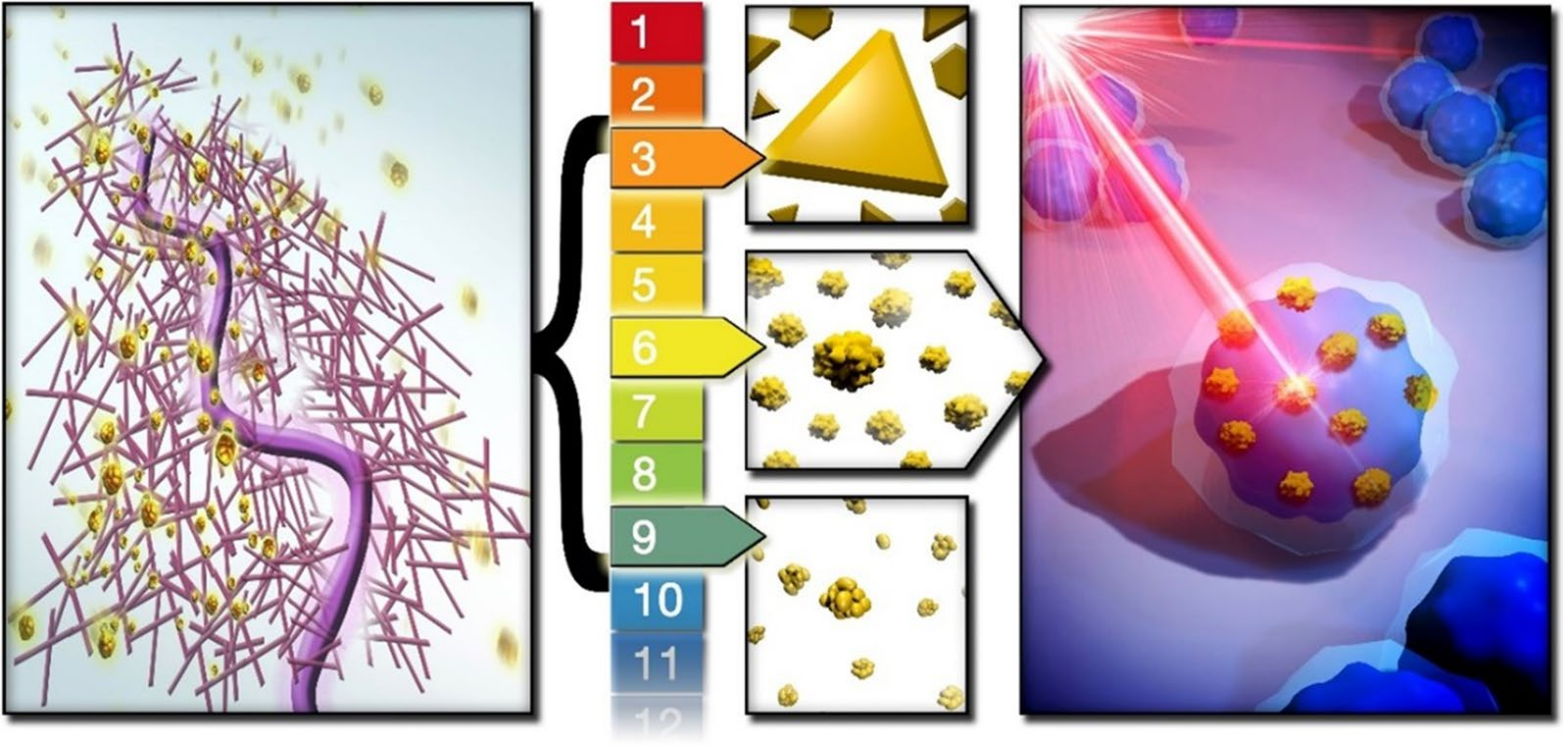
Schematic showing that AuMu nanostructures with different shapes, sizes, and aggregation states can be synthesized in the presence of different PGM conformations induced by an acidic, neutral, and alkaline environment. The PT properties of the materials formed allow localized heating to treat bacterial infections when exposed to near-infrared laser irradiation

Here, we demonstrate a novel one-pot *programmable* green bio-assisted synthesis that facilitates the formation of various types of Au structures, all synthesized in situ in PGM (AuMu) in a controlled way. The structures vary three orders of magnitude in size (from nm- to µm- regimes), structure (spherical nanoparticles, coral-like aggregates, and micro-plates), and aggregation density. We perform comprehensive kinetic and growth analyses that shed new light on the complex process's mechanism to understand this phenomenon better. We further demonstrate the excellent photothermal properties of the AuMu and explore their potential as biocompatible bactericidal nanomaterials (Scheme 1).

## Results and discussion

### pH-dependent synthesis

**Fig. 1:**
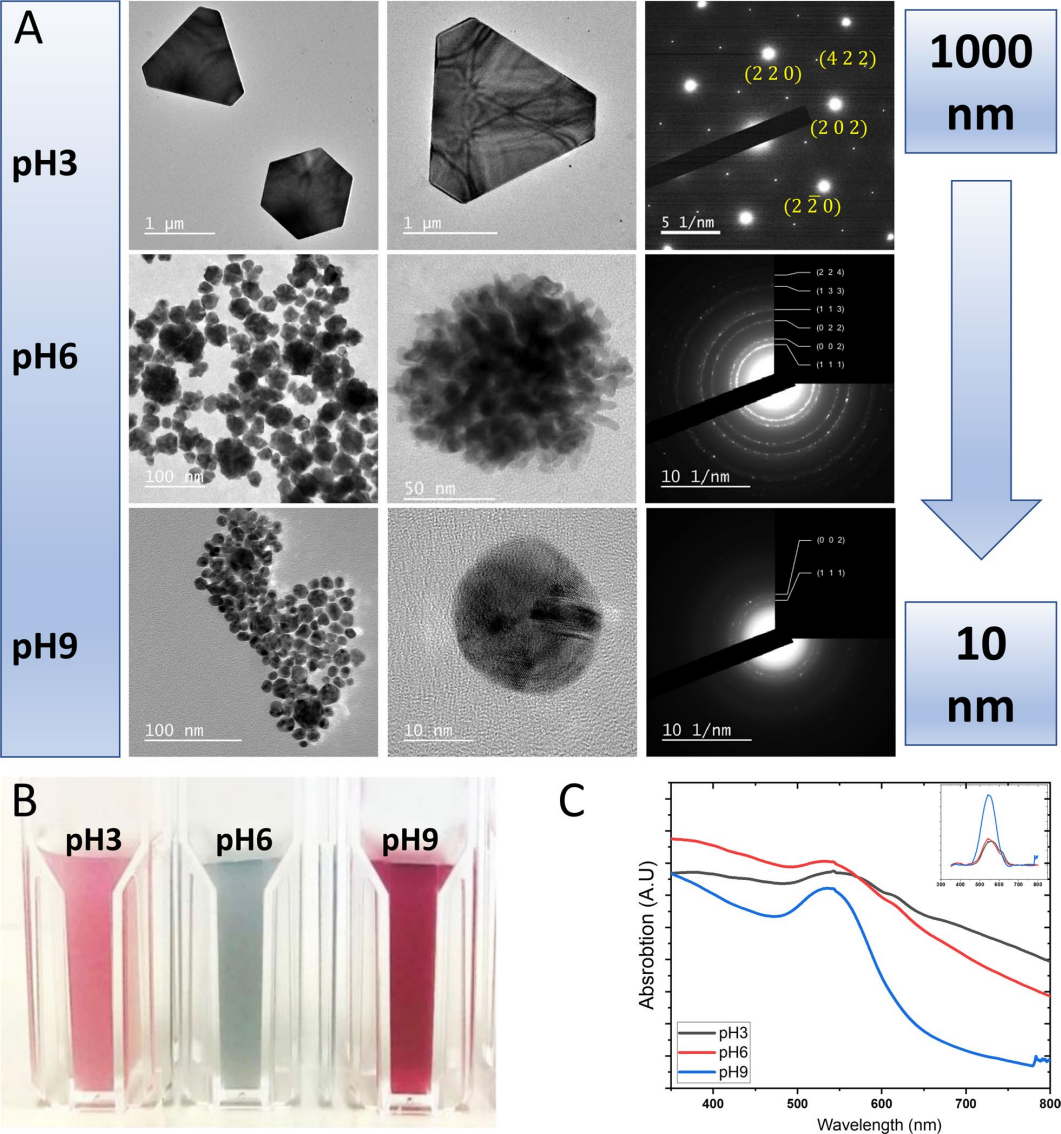
A Bright-field TEM images (two left columns) and selected area diffraction patterns (SAED, right) of AuMu nanostructures synthesized at pH 3, pH 6, and pH 9, forming micron-sized crystals (triangles and hexagons), mainly coral-shaped nanoparticles and spherical nanoparticles, respectively. In all cases, individual Au particles were perfectly crystalline apart from the presence of planar defects. B. Optical image of the Au@PGM solutions. C. UV–vis spectra of the solutions. Inset: UV–vis spectra obtained after background substruction highlighting the plasmonic peak

To study the structural properties of AuMu at different conditions, we performed the synthesis at pivotal points in which PGM exhibits the most pronounced conformational changes (pHs of 3, 6, and 9). The resulting AuMu were characterized using Transmission Electron Microscopy (TEM, Fig. 1A, Additional file 1: Fig. S1). Figure 1A shows various types of AuMu exhibiting clear characteristics of face-centered cubic structure (FCC) [60, 61]. While the same crystal type was obtained at all pHs, the shape and size of the AuMu obtained were substantially different. At pH 3, µm-sized Au platelets were formed, displaying triangular, truncated-triangular, and hexagonal flakes and some scattered population of sub-50 nm-sized spherical particles. While the hexagonal shape is attributed to the equilibrium crystal shape of the {111}-oriented flake, the triangular and truncated triangular shapes are transitional, indicating an ongoing growth process. [62, 63]. Previous observations on epitaxially grown Si [62] or other 2D crystals [64] have convincingly shown that hexagonal shapes were established upon cessation of atoms' growth dissolution from the triangle apexes towards a truncated triangular shape and eventually to a compact hexagon. This shape transition is driven to equilibrium by edge and corner energy considerations once no more free atoms are available for further flake growth. The higher energy of the less coordinated atoms at the apex sites drives their detachment from the receding corners into progressively truncated side edges. An opposite process takes place if/when growth is renewed: the {110} bounding edges advance radially outwards along respectively perpendicular {112} directions, leading to a fully equilateral hexagon if all the edges have the same energy (or slightly different edge lengths, if not). While the process demonstrated here is quite different from epitaxial growth, the similarities in transitional and equilibrium shapes are apparent. As shown in Additional file 1: Figures S2A and B, both shape types' simultaneous presence evidences the dynamic nature of the AuMu@pH3 flake formations. In addition to the expected hexagonal single crystal, selected area electron diffraction (SAED) pattern at this pH, formed by {220} spots along a {111} zone axis, 1/3{422}-type reflections forbidden by FCC diffraction selection rules (red circles) were clearly visible (Fig. 1A). Such forbidden reflections have been often observed in FCC 2D flakes obtained by wet chemical methods and attributed to planar defects lying parallel to the {111} surface plane [62] and taking an active role in the formation and growth of the {111}-oriented flakes [64].

In AuMu@pH6 (Fig. 1, Additional file 1: Figure S1), the dominant morphology was coral-shaped clusters ranging from 10 to 50 nm. At this pH, the SAED pattern is also fully consistent with FCC Au reflecting planes; however, this time, they exhibit a ring rather than a spotted pattern. This was expected given the polycrystalline nature of the almost randomly oriented tiny crystals (some spots lying on the Debye rings indicated a certain extent of preferred orientation).

AuMu@pH9 were less randomly oriented (Fig. 1, Additional file 1: Figure S1) than AuMu@pH6. Increased spottiness and the distribution of a discontinuous intensity along the FCC SAED rings were evident due to the globules' non-negligible texture. Additionally, planar defects were clearly visible in some of these structures.

Optical images (Fig. 1B) of the solutions were also taken, showing different colors according to their optical properties. The corresponding UV–Vis (Fig. 1C) spectra show that all the structures exhibit a strong plasmon peak at around 500 nm, while AuMu@pH3 and AuMuc@pH6 showed an additional peak at 620 nm, indicating on a broad population of structures.

### Growth and kinetics mechanisms

**Fig. 2:**
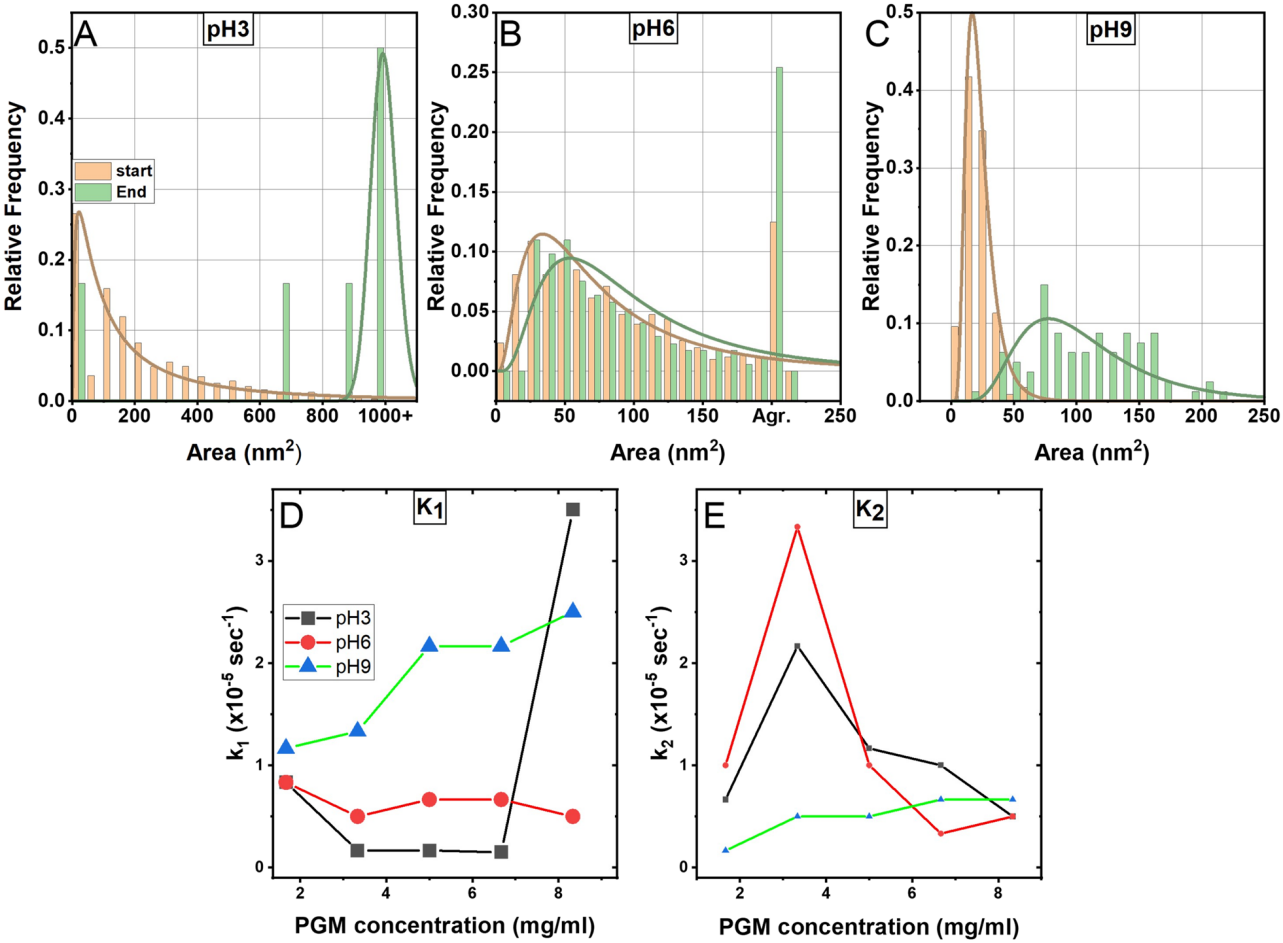
Growth and kinetics study. **A**–**C** Size distribution study obtained from TEM data of sample in which [PGM] = 30 mg/ml and at timelapse of t = 120 min and t = 330 min Kinetics and size distribution analysis. **D** Evolution of k_1_ and k_2_ **E** as a function of PGM concentration

Further insight into the growth mechanisms was achieved by time-dependent and size-distribution analysis using time-lapse TEM measurements followed by kinetic studies of the reaction obtained by UV–Vis spectroscopy (Fig. 2).

It is rather challenging to determine all the details of growth mechanisms that may have taken place at all the concentrations, pH levels, and reaction stages in the current experimental design, even after a scrupulous study of the time-lapse TEM imaging. It is not straightforward even for less complicated model experiments because it is difficult to separate factors affecting size distributions and their correct measurements in bio-assisted synthesis systems [65, 66]. In many systems, the late growth stages (where free species are no longer available for new nucleation events) are governed by the Gibbs–Thomson tendency to reduce vapor pressure/chemical potential around curved boundaries [67]. Lifshitz, Slyozov, and Wagner (LSW) have demonstrated that particles coarsened by the Ostwald ripening mechanism exhibit characteristic negatively skewed size distributions (LSW functions) [68, 69]. Dynamic coalescence, where clusters grow due to fusion between diffusing particles bumping onto each other, is often characterized by positively skewed size distributions that can be fitted with a log–normal function. In reality, however, it is unclear under what conditions coalescence events begin to prevail overripening and whether these events are sufficient to tangibly skew the size distribution.

In our case, dynamic coalescence was the prevailing particle growth mechanism, certainly at the early evolution stages and most likely at the late growth stages (Fig. 2A-C). This is quite apparent from the aggregates' fractal shapes formed by the coagulation of small moving particles at higher pH. Even under the lowest pH, large polygonal flakes are shown to keep growing by attachments of small mobile particles originating from the solution to the polygonal flake boundaries (Fig. 1, 2A-C, Additional file 1: Figure S2). Again, positively skewed size distributions in the case of AuMu@pH3 (Fig. 2A left) support dynamic coalescence as a leading cluster growth mechanism throughout the entire growth duration. A certain degree of competition between ripening and coalescence at higher pH in more highly concentrated solutions cannot be entirely excluded, judging from occasionally revealed denuded zones around large clusters (and multimodal size distribution plots) (Fig. 2A) and given the complexity of the process.

A complementary perspective on the growth mechanism can be obtained from the measurements of the concentration-dependent kinetics. In the framework of the current study and experimental conditions, the concentrations of PGM ([PGM]) at the selected range of pHs were held in excess with respect to Au ions ([Au]); thus, the limiting factor in the reaction was the amount of [Au] ions [70–72]. At these conditions, a pseudo-first-order reaction model can be applied, which assumes that: [PGM], [Cys] = constant >  > [Au]. Thus, the reaction rate, *r*, can be rewritten as:

$$r\, = \,k^{\prime}\;\left[ {{\text{PGM}}} \right]\;[{\text{Au}}]$$

Where *k'* is the second-order reaction rate constant. Since [PGM] = constant, the rate equation can be simplified to give:

$$r\, = \,k\,[{\text{Au}}]$$

Where *k* = *k'* [PGM].

We evaluate *k*_*1,2*_ following a previously demonstrated analysis in which the energy shift of the Au plasmonic absorbance peak (A_t_) was traced using UV–Vis at different time points [67]. In this type of analysis, it can be assumed that similar processes can be reasonably represented by two rate constants: *k*_*1*_ and *k*_*2*_ [68, 69]. *k*_*1*_ corresponds to the initial stages of the nucleation and growth in which Au ions diffuse towards the PGM and form nucleation centers near the PGM’s folded or unfolded hydrophobic domains. Once nucleation commences, the second stage of the reaction occurs (represented by *k*_*2*_)*,* in which the growth of the AuMu in their final form takes place.

**Fig. 3:**
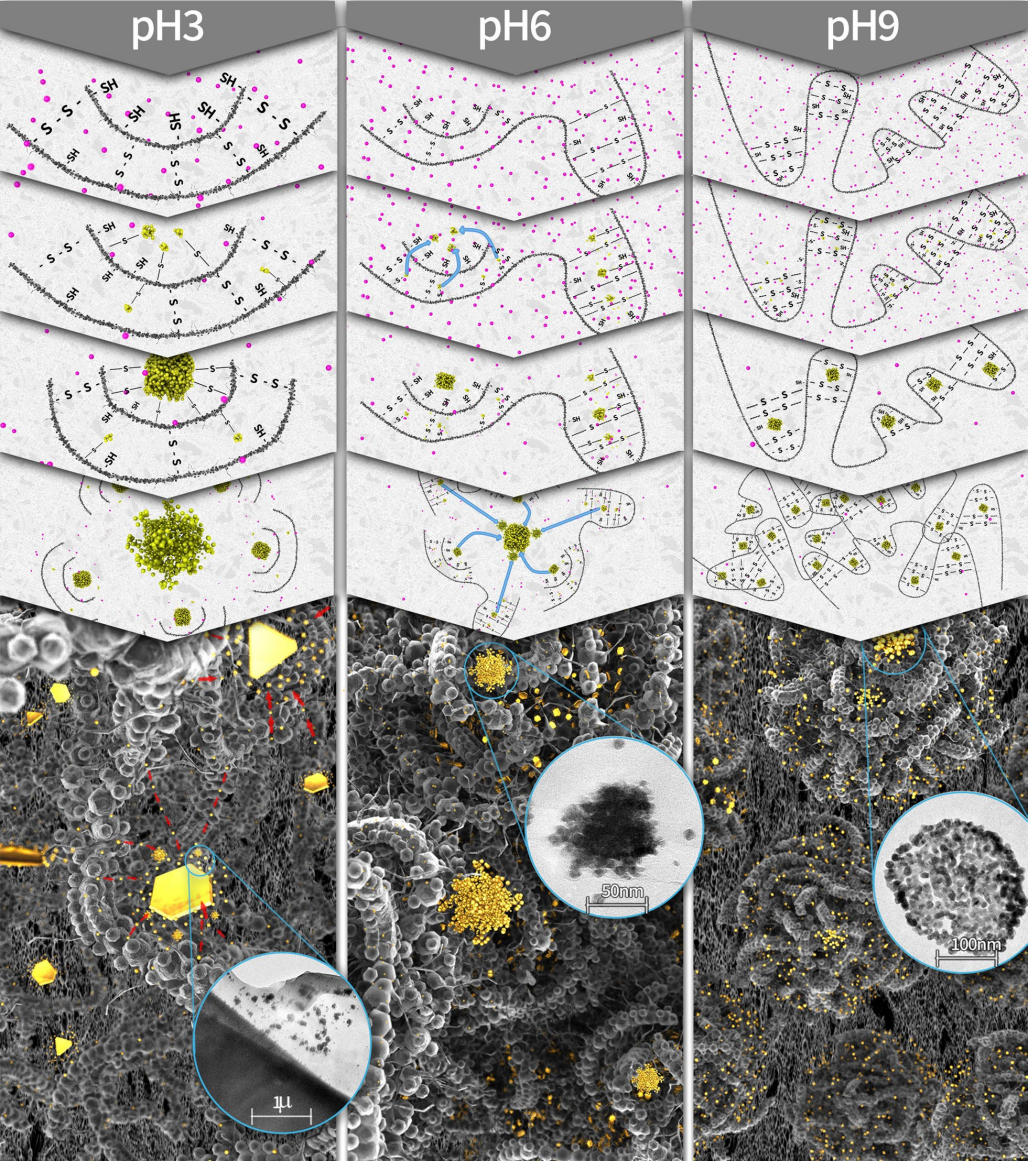
The suggested mechanism of formation of the AuMu nanostructures. At pH 3, the nucleation step takes place at the Cys sites exposed within the unfolded PGM chains. Next, coalescence between the particles occurs, and the resulting structures then form triangular and hexagonal structures. At pH 9, the closed pockets in the PGM folded form dictate only nanoparticles' formation since diffusion at these conditions is limited. At pH 6, where the PGM is partially folded, coral-like structures are formed by coalescence of the small nanoparticles at the folded pockets, with the larger ones nucleated at the unfolded sites. Inserts: TEM images of formed nanoparticles and nanostructures at different pHs)

Wadhwani et al. 69 demonstrated that *k*_*1*_ and *k*_*2*_ can be obtained from the slope of the linear part of the log (A_f_-A_t_) *vs*. time, where A_f_ is the value of the maximal peak at the end of the reaction and A_t_ is the maximal peak obtained in different time points (Fig. 2D, E, and Additional file 1: Figure S3 for a detailed analysis). First, we observe that *k*_*1*_ increases with PGM concentration at pH 9, while at pH 6, it remains constant (Fig. 2D). At pH 3, a sharp increase in large concentration is observed. Up to this turning point, it is evident that *k*_*1*_@pH 9 > *k*_*1*_@pH 6 > *k*_*1*_@pH 3. This finding indicates that the nucleation process is faster at the PGM's closed pockets (pH 9), where the Cys moieties do not form intermolecular interactions and are close to each other [41, 42, 45]. This leads to more sites available for reducing the Au ions than in the open PGM structure formed at pH 3 and in low concentrations. At pH 3, the sharp increase of *k*_*1*_ above a 6.7 mg/mL concentration could be correlated with the well-known sol–gel structure formation of Mucins at high concentrations (Fig. 3) [41, 42]. When reaching this form, the number of intramolecular bonds are reduced [45, 47, 49] while the number of free Cys groups capable of chemically reducing Au ions increases, thus increasing k_1_. At pH 6, most of the PGM's pockets are in their folded states; thus, k_1_ does not change dramatically.

In the next growth phase (roughly represented by *k*_*2*_, Fig. 2E), the conformation of PGM globules and the degree of gelation determines the final shape and size of the resulting AuMu structures. Since the PGM's sol–gel density increases at larger concentrations, limiting the Au particles' diffusivity across the PGM protein units. This phenomenon is demonstrated at pH 3 and 6, in which a decrease of *k*_*2*_ takes place from the critical concentration of 3.3 mg/mL_*.*_.

Figure 3 illustrates a suggested mechanism based on the kinetics and growth studies.

### Evaluation of the photothermal effect of AuMuc NPs: a potent antibacterial nanomedicine

Given the increasing interest in programming Au nanostructures that present tunability in shape and size, biocompatibility and non-toxicity, and ability to respond to an external trigger, we explored the use of AuMu as antibacterial PT agents.

**Fig. 4:**
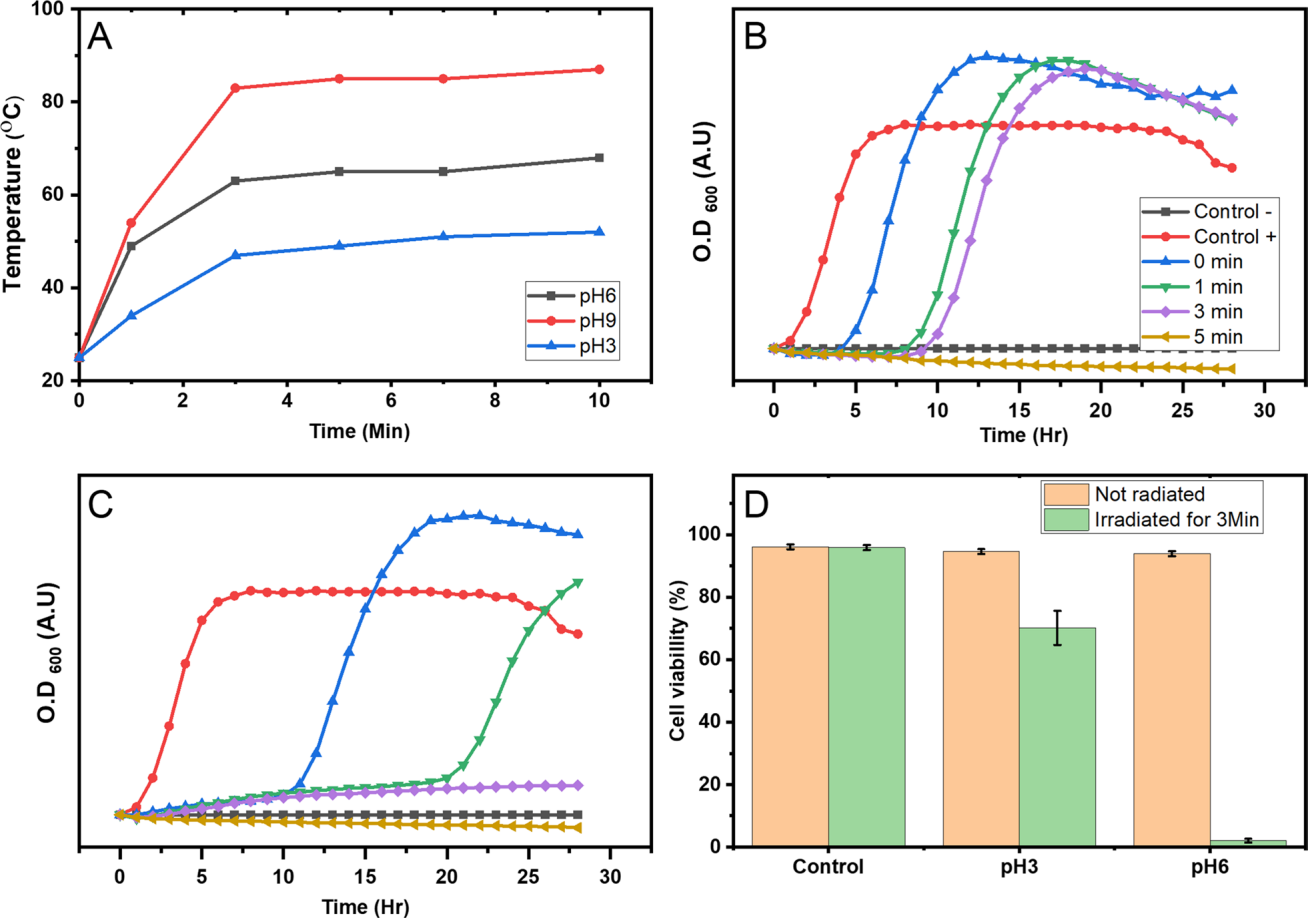
**A** Heating performance of AuMu nanostructures synthesized at pH 3, 6, and 9 (PGM concentration of 8 mg/ml) under irradiation with an 808 nm NIR laser at 1.25 W/cm^2^. **B–C** Bacterial growth following irradiation of the MRSA-infected TS broth with the NIR laser for 0, 1, 3, and 5 min after incubation with AuMu@pH3 (B) and AuMu@pH6 (**C**). **D** Cell viability of human foreskin fibroblasts (HFF) cells was measured through a fluorescein diacetate (FDA) metabolism assay after incubation with AuMu and irradiation for 3 min in order to simulate the treatment damage to the healthy skin surrounding tissue

For this purpose, we evaluated the photoactivity of the AuMu synthesized at different pHs and PGM concentrations after exposure to NIR (λ = 808 nm) against MRSA bacteria as proof of concept (Fig. 4A, Additional file 1: figure S4).

The analysis indicates that the PT effect varies with the synthesis parameters (representative measurements in Fig. 4A and complete study in Additional file 1: Figure S4). The findings indicate a direct correlation between the concentration of the reducing agent (PGM), reaction time, particle shape and size, and maximum heating capacity (Additional file 1: Figures S1, S4). Notably, no direct correlation was found between the Au's typical absorption plasmonic peaks (~ 500–600 nm) and the material's PTs' activity. This observation could be attributed to the heat generated by light scattering in particles located close to each other, promoting the heating through the Anderson Localization mechanism [57, 58]. Indeed, small and more dispersed AuMu@pH9 particles synthesized in all concentrations exhibit weaker PT properties than the coral-shaped AuMu@pH6 particles and the crystalline AuMu@pH3 ones (Additional file 1: Figure S4). It can be concluded that the coral-shaped AuMu@pH6 particles exhibit the highest temperature increase with increased PGM concentration, probably due to increased light scattering from these structures.

Assessment of the AuMu as a bactericidal agent against methicillin-resistant MRSA in vitro was carried out by simulating an infected wound environment (Fig. 4B, C). The infected wound environment was simulated by nutrient Tryptic Soy (TS) broth inoculation with MRSA overnight cultures and mixed with the two different AuMu at equal mass concentrations. AuMu@pH3 and AuMu@pH6 were used for this proof-of-concept experiment as they produce the most rapid temperature increase and highest heating temperatures. The prepared formulations were irradiated with a NIR laser at 1.25 W/cm^2^ for 0, 1, 3, and 5 min in order to determine the minimum inhibitory duration for laser irradiation.

The curves in Fig. 4B, C represent bacterial growth following the infected TS broth irradiation with the NIR laser for 0, 1, 3, and 5 min. This was compared to the negative control, where the TS broth was kept sterile, and the positive control, where the overnight colonies were allowed to grow without the addition of AuMu or laser irradiation. Since AuMu@pH6 demonstrates a steeper heating profile than AuMu@pH3 upon laser irradiation, bacteria grown with AuMu@pH6 were prevented from reaching logarithmic growth with as little as 3 min of laser irradiation, while AuMu@pH3 required 5 min of heating in order to completly suppress MRSA growth over the period of 28 h. In each case, lower irradiation durations caused delays in the log-growth phase, but no complete growth suppression was observed.

Interestingly, the mere inclusion of the AuMu into the bacterial broth resulted in a delayed log-growth compared to the positive control with an AuMu@pH9 delay of 9 h relative to the positive control. This has been attributed to the mild antibacterial properties of Au nanoparticles ^56^ and not to the presence of mucin since mucin alone (1 mg/mL) shows no negative effects on bacterial growth (Additional file 1: Figure S5).

In order to demonstrate that this hyperthermal behavior of the AuMu@pH6 and AuMu@pH3 is a result of the shape of the nanostructures and not a plasmon resonance effect due to absorption at 550 nm, we tested commercially available biotin-capped 70 nm spherical nanoparticles suspended in 1 × PBS at a concentration of 6 × 10^–12^ M under NIR laser irradiation. It is evident from Fig. 4A that no significant temperature increase is observed even after 10 min of irradiation except for a few degrees increase in solution temperature. Similarly, the same concentration of biotin-capped Au nanoparticles was tested against 10^8^ CFU of MRSA in TS broth under different durations of laser irradiation. Additional file 1: Figure S6 shows that laser irradiation of the inoculated broth at durations of 1, 3, and 5 min yielded the same results as no irradiation at all and the logarithmic growth phase of the MRSA was reached after 1–2 h of incubation in all the cases.

The inclusion of the biotin-capped Au nanoparticles with the bacteria without irradiation did not delay the onset of log growth compared to what was observed for AuMu@pH3 and AuMu@pH6. We attribute this observation to the fact that the commercial Au nanoparticles are surrounded by biotin, and therefore, the Au nanoparticles were effectively screened from interacting with bacterial cells directly while AuMu are less shielded by PGM; therefore, the surface of Au remains exposed to interact with bacteria directly.

Furthermore, to ensure that exposure to AuMu only kills bacteria in the wound but does not drastically affect skin cells, we tested the effect of AuMu formulations on human skin cells' viability. For this, both AuMu@pH3 and AuMu@pH6 formulations were first incubated with HFF to ensure they are non-toxic to healthy cells. Figure 4D shows HFF viability rates as a function of incubation with the AuMu at standard conditions for 48 h. Furthermore, some of the cell-AuMu mixtures were also irradiated with the NIR laser for 3 min before incubation to simulate the optimal bactericidal conditions established in the previous section and measure HFF resilience to the irradiation and elevation in temperature. As expected, cells incubated without the presence of AuMu formulations as control cultures exhibited ~ 96% viability even under laser irradiation for 3 min, confirming the non-invasiveness of NIR irradiation. Cells incubated with both AuMu@pH3 and AuMu@pH6 without any laser irradiation also showed no significant decrease in viability, with both displaying viability rates of ~ 94% each. This confirms the non-toxicity and biocompatibility of AuMu. However, under 3 min laser irradiation, HFF cells incubated with AuMu@pH6 caused a dramatic decrease in cell viability, with only 2% of the cells surviving. This was due to the extreme temperature increase observed for AuMu@pH6 where 3 min of irradiation caused a ~ 60 °C increase in 1 mL of solution.

On the other hand, the mixture containing the AuMu@pH3 formulation exhibited ~ 75% cell viability after 3 min of irradiation compared to 94% for the control cultures. Under these conditions the AuMu@pH3 formulation still resulted in a considerable 9 h growth delay of MRSA (Fig. 4B). Even though a 25% reduction in cell viability is not ideal, this formulation can be used to suppress the growth of MRSA in wounds, for example, by applying the formulation onto the exposed wound and applying periodic bursts of NIR laser energy. Furthermore, given that the heating effect is localized, the addition of bio-targeting moieties to the surface of the AuMu might increase bacterial attachment selectivity and therefore decrease the effective dosage and irradiation time required to suppress MRSA growth.

## Conclusions

In conclusion, we investigated PGM's reduction capabilities to produce various Au nanostructures by utilizing this biomolecule as a sole reducing and capping agent in a one-pot green synthesis approach. We showed that protein conformation induced by pH could drastically affect the nanostructure size, shape, aggregation, and photothermal properties, while PGM concentration influences the reaction kinetics and growth. We optimized and used the photothermal properties of produced AuMu for antibacterial application against Methicillin Resistant S.aureas (MRSA) bacteria. AuMu nanoparticles were tested for their possible toxicity on healthy skin cells to determine which particles produce minimal collateral damage to surrounding cells while still having the highest potential to inhibit or disrupt bacterial growth. Based on our results, the tunability of the PT properties of AuMu can be leveraged as stand-alone or additive antibacterial material for existing or new wound dressing material as it possesses both passive and active antibacterial properties and minimal cellular toxicity.

## Methods

### AuMu NP synthesis

50 mg of PGM (Sigma Aldrich) was mixed with 5 mL gold salt (2.5 mM of AuHCl_4_, STREM). The mixture was stirred until the salt was fully dissolved. Next, 2.5 mL of glycine buffer (200 mM pH = 3, 6, and 9) were added to the solution, and the mixture was further stirred for an additional hour for pH stabilization. Fine-tuning of the buffer pH was done by titration of hydrochloric acid or sodium hydroxyl. After the solution was stabilized, it was purged with nitrogen gas and stirred at room temperature in the dark for 72 h. Alternatively, the solution was heated to 70 °C to obtain similar results in a shorter time (< 30 h) for the kinetics and growth study. Next, the sample's residual gold ions were removed by a dialysis (14 kDa, Sigma Aldrich), resulting in AuMu exhibiting a molar weight of larger than 12,400 kDa.

### Kinetics study

The kinetics study of AuMu formation was performed using a solution of PGM with concentrations of 1.7 mg/mL, 3.3 mg/mL, 5 mg/mL, 6.7 mg/mL and 8.3 mg/mL. The Au ions concentration was kept constant (0.6 mg/mL in pH 3 and pH 9, and 0.42 mg/mL in pH 6) throughout the reaction. At time intervals, an aliquot of each sample was analyzed by High-Resolution Transmission Electron Microscope (HR-TEM) and UV–Vis to monitor the synthesis progress.

### UV–Vis characterization

UV–Vis spectrometer measurements were carried out using a Cary 5000 high-performance UV–VIS-NIR spectrometer operated at a resolution of 5 nm. Each AuMu solution taken at different reaction time intervals was diluted appropriately with pH glycine buffer and placed in quartz cuvettes with a beam length of 10 mm. The spectra of each sample were measured from 250 to 1000 nm.

### HR-TEM characterization

A JEOL 2100F transmission electron microscope was used at 200 kV for the imaging and structural analysis by electron diffraction of the samples. AuMu were diluted 20 × in Milli-Q water and were deposited and dried on a carbon-coated copper grid (PST ProSciTech).

For the kinetics study, TEM images of AuMu were obtained over period of 900 min minutes into the reaction with time interval range 30–120 min in Philips Tecnai F20 ST at an accelerating voltage of 200 kV. The samples were applied on copper TEM grids by dip coating and dried under a fume hood.

### Photothermal measurements of AuMu

AuMu samples were irradiated with an 808 nm continuous-wave diode laser at a power density of 1.25 W/cm^2^ in water for 1, 3, 5, 7, and 10 min. The temperature of the solutions was measured at each time point using a thermocouple.

Similar experimental conditions were applied to samples prepared for kinetic study (irradiation time of 3 min) to measure and correlate the effect of AuMu formation on their photothermal properties.

### Bacterial studies

AuMu@pH3/pH6 formulations were prepared at a 1.7 × 10–2 mM Au concentration in TS broth by first centrifugation and removal of buffer solution, followed by the addition of TS broth and resuspension of the AuMu using ultrasonication. Methicillin-resistant *S. aureus* (ATCC 43300) was obtained from frozen cultures and streak-plated onto pre-prepared TS agar plates and allowed to incubate at 37 ˚C overnight. One colony was picked and used to inoculate 10 mL of Tryptic Soy (TS) broth. The inoculated broth was then incubated overnight at 37 ˚C to generate overnight cultures of each bacterial strain with a concentration of 10^8^ CFU. In 96 well plates, 100 µl of the AuMu@pH3/pH6 formulation was added to each well, and 100 µl of the bacterial overnight culture. Each well plate was then irradiated with an 808 nm collimated laser beam (Changchun Optoelectronics diode laser, MDL-H-808, PSU-H-LED driver) at 1.25 W/cm^2^ for durations of 0, 1, 3, and 5 min. Following the irradiation, the AuMu@pH3/pH6 bacterial mixtures were then transferred to 48 well plates and diluted further with 1.8 mL of TS broth to form 2 mL of solution. The plates were then placed in a pre-warmed (37 ˚C) plate reader, and bacterial growth was monitored at 600 nm for 28 h at 1 h intervals.

### Cell viability

HFF were grown and maintained at 37 ºC with 5% CO_2_ in Dulbecco's modified Eagle's medium (DMEM) supplemented with 10% fetal bovine serum (FBS, Sigma), 2 mM GlutaMAX (Life Technologies), 100 U/mL penicillin, and 100 µg/mL streptomycin (Life Technologies), for 2–3 d until they were 70%–80% confluent. The HFF cells were seeded onto a 96-well plate (BD Falcon) at a density of 2 × 10^4^ cells/mL in fresh complete DMEM per well. The AuMu-NPs were sterilized by antimycotic antibiotic 4X solution (Sigma) in sterile PBS for 5 min and washed twice in sterile PBS to remove the antimycotic antibiotic solution. After 1 d, HFF cells were incubated with AuMu-NPs for 10 min at a concentration of 1.7 × 10^–2^ mM (Au) at 37 ºC with 5% CO_2_. Following incubation, cells with AuMu-NPs and without were irradiated with an 808 nm collimated laser beam (Changchun Optoelectronics diode laser, MDL-H-808, PSU-H-LED driver) at 1.25 W/cm^2^ for durations of 3 min as described. The cells were incubated at 37 ºC with 5% CO_2_ for 48 h before cell viability measurements.

The viability of cells was quantitatively assayed by a lactate dehydrogenase (LDH) assay kit (Abcam) according to the manufacturer's recommendations. Briefly, after 48 h incubation with AuMu-NPs, 100 µl of the cell suspension was centrifuged at 600 × g for 10 min, and the supernatant was collected to carry out the assay. 100 µl of each sample was transferred to each well into a flat-bottomed 96-well plate (Nunc). 100 µl of LDH reaction mix was added to each well. The wells were gently mixed and were further incubated at room temperature for 30 min. The absorbance at 450 nm was measured with a microplate reader, and all cultures were performed in triplicates (*n* = 3).

## Supplementary Information

**Additional file 1: Figure S1.** Initial and finalTEM images of AuMu biosynthesis in various concentrations of PGM (10mg, 20mg, 30mg, 40mg and 50 mg in solution volume of 7 mL for pH 3 & pH 9 and 6mL for pH6) and at pH3, 6 and 9. **Figure S2.** TEM images of reaction kinetic phases of the AuMuN Pbiosynthesis under pH3 environment.A) The synthsis starts (phaseone) by the formation of spherical particles under *k 1*reaction rate.B) The fusion of spherical particles into complex triangular particles under *k2 *reaction rate in phase two. **Figure S3.** Kinetic analysis from the UV-V is data obtained from synthesis reaction at different PGM concentrations at different reaction times. The rate constant k was obtained from the slope of graph of logarithm of maximum AuNP plasmonic absorption peak at the final stage of synthesis reaction minus maximum AuMu plasmonic absorption peak at the beginning of the reaction infront of the reaction time [5, 45, 46]. The indication of time when the reaction began is the transition of the color from the Au ions + PGM solution: from pale yellow to pink (pH3), bluish (pH6) or red (pH9). **Figure S4.** Photothermalproperties of the AuMu complexes biosynthesized at different PGM concentration and pH.A) 1.7mg/mlPGM, B) 3.3mg/ml PGM, C) 5mg/mlPGM, D) 6.7mg/mlPGM, E) 8.3mg/ml PGM. The data show the maximum temperature reached under NIR laser irradiation *vs. *time of the reaction (errorof±1ºCand±1 min). **Figure S5.** MRSA growth in TS-broth over time in the presence of 1mg/mL of pure mucin. The MRSA inoculations were prepared at different starting concentrations (105,106, and 107) with control representing a sterile solution. **Figure S6.** MRSA growth vs. 808 nm laser irradiation for 0, 1, 3, and 5 min in the presence of 6x10-12 MauNPs (70 nm, biotinterminated).

## Data Availability

All data generated or analysed during this study are included in this published article [and its supplementary information files].

## Abbreviations

Cys: Cysteine
PGM: Porcine gastric mucin
Au: Gold
NP: Nanoparticles
PT: Photothermal
MRSA: *S. aureus*
NIR: Near-infrared
Mu: Mucin
TEM: Transmission electron microscopy
FCC: Face-centered cubic structure
SAED: Selected area electron diffraction
LSW: Lifshitz, Slyozov, and Wagner
HFF: Human foreskin fibroblasts cells
FDA: Fluorescein diacetate
TS: Tryptic soy
LDH: Lactate dehydrogenase
