# Supplementary material for: From nanoparticles to crystals: one-pot programmable biosynthesis of photothermal gold structures and their use for biomedical applications
Source: J Nanobiotechnology. 2022 Nov 16;20:482. doi: 10.1186/s12951-022-01680-7 (PMC9670439; doi:10.1186/s12951-022-01680-7)
Supplement: Supplementary file 1 — Additional file 1: Figure S1. Initial and finalTEM images of AuMu biosynthesis in various concentrations of PGM (10mg, 20mg, 30mg, 40mg and 50 mg in solution volume of 7 mL for pH 3 & pH 9 and 6mL for pH6) and at pH3, 6 and 9. Figure S2. TEM images of reaction kinetic phases of the AuMuN Pbiosynthesis under pH3 environment.A) The synthsis starts (phaseone) by the formation of spherical particles under k 1reaction rate.B) The fusion of spherical particles into complex triangular particles under k2 reaction rate in phase two. Figure S3. Kinetic analysis from the UV-V is data obtained from synthesis reaction at different PGM concentrations at different reaction times. The rate constant k was obtained from the slope of graph of logarithm of maximum AuNP plasmonic absorption peak at the final stage of synthesis reaction minus maximum AuMu plasmonic absorption peak at the beginning of the reaction infront of the reaction time [5, 45, 46]. The indication of time when the reaction began is the transition of the color from the Au ions + PGM solution: from pale yellow to pink (pH3), bluish (pH6) or red (pH9). Figure S4. Photothermalproperties of the AuMu complexes biosynthesized at different PGM concentration and pH.A) 1.7mg/mlPGM, B) 3.3mg/ml PGM, C) 5mg/mlPGM, D) 6.7mg/mlPGM, E) 8.3mg/ml PGM. The data show the maximum temperature reached under NIR laser irradiation vs. time of the reaction (errorof±1ºCand±1 min). Figure S5. MRSA growth in TS-broth over time in the presence of 1mg/mL of pure mucin. The MRSA inoculations were prepared at different starting concentrations (105,106, and 107) with control representing a sterile solution. Figure S6. MRSA growth vs. 808 nm laser irradiation for 0, 1, 3, and 5 min in the presence of 6x10^-12^ MauNPs (70 nm, biotinterminated). [file 12951_2022_1680_MOESM1_ESM.pdf]

Supporting Information

From nanoparticles to crystals: one-pot programmable biosynthesis of photothermal gold structures and their use for biomedical applications.

Roman Nudelman, Hashim Alhmoud, Bahman Delalat, Ishdeep Kaur, Anastasia Vitkin, Laure Bourgeois, Ilan Goldfarb, Anna Cifuentes-Rius, Nicolas H. Voelcker*, Shachar Richter**

R. Nudelman, A. Vitkin, Prof. I. Goldfarb, Prof. S. Richter
Department of Material Science and Engineering, Center for Nano-science and Nano-technology, Tel-Aviv University, Tel-Aviv 69978, Israel.
E-mail: srichter@tauex.tau.ac.il

Dr. H. Alhmoud, I. Kaur, Prof. N. H. Voelcker, Dr. A. Cifuentes-Rius
Monash Institute of Pharmaceutical Sciences, Monash University, Parkville Campus, 381 Royal Parade, Parkville VIC 3052, Australia
E-mail: anna.cifuentesrius@monash.edu, nicolas.voelcker@monash.edu

Dr. B. Delalat, Prof. N. H. Voelcker
CSIRO Manufacturing, Bayview Avenue, Clayton, Victoria 3168, Australia

A/Prof. L Bourgeois
Monash Centre for Electron Microscopy, Department of Materials Science and Engineering, Faculty of Engineering, Monash University, Clayton Campus, 10 Innovation Walk, Clayton VIC 3168, Australia

Prof. N. H. Voelcker
Melbourne Centre for Nanofabrication, Victorian Node of the Australian National Fabrication Facility, 151 Wellington Road, Clayton, Victoria, 3168, Australia

Keywords: protein-templated synthesis, gold nanoparticles, mucin, green synthesis, laser irradiation, antibacterial activity

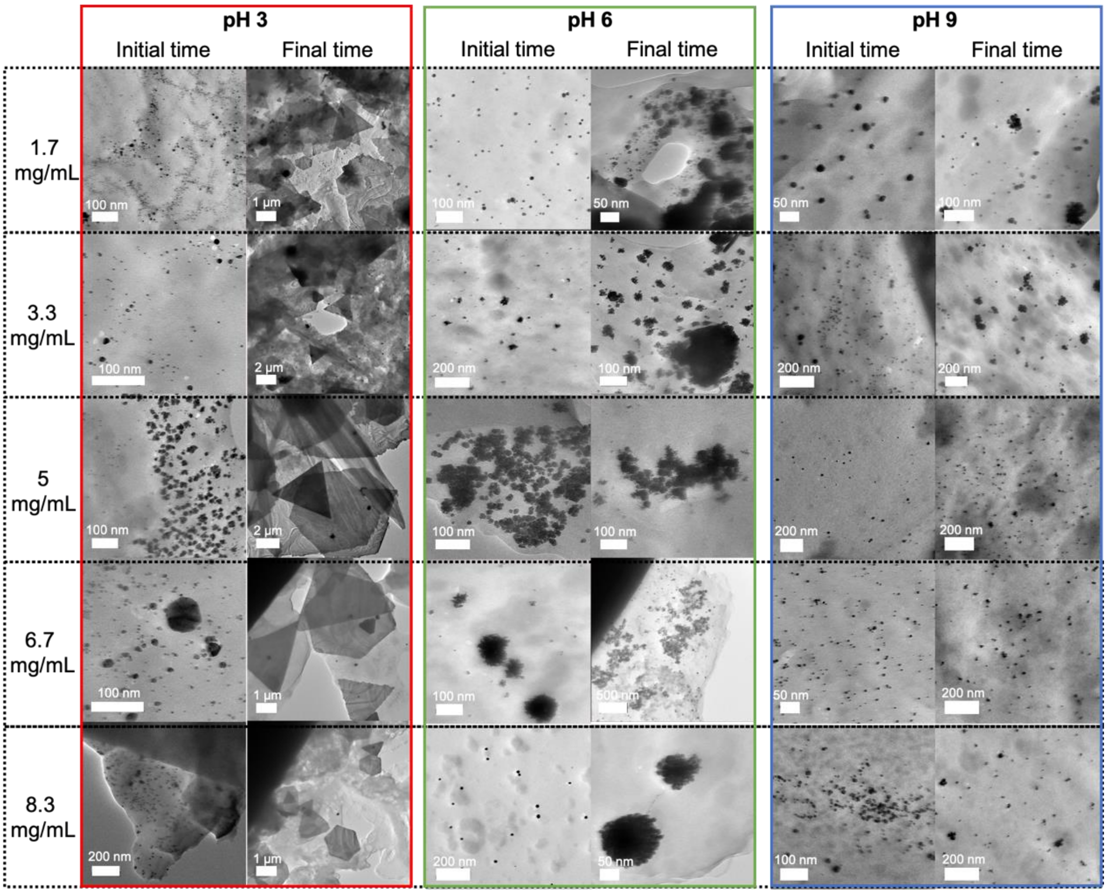

Figure S1. Initial and final TEM images of AuMu biosynthesis in various concentrations of PGM (10 mg, 20 mg, 30 mg, 40 mg and 50 mg in solution volume of 7 mL for pH 3 & pH 9 and 6 mL for pH 6) and at pH 3, 6 and 9.

TEM analysis of the reaction kinetics

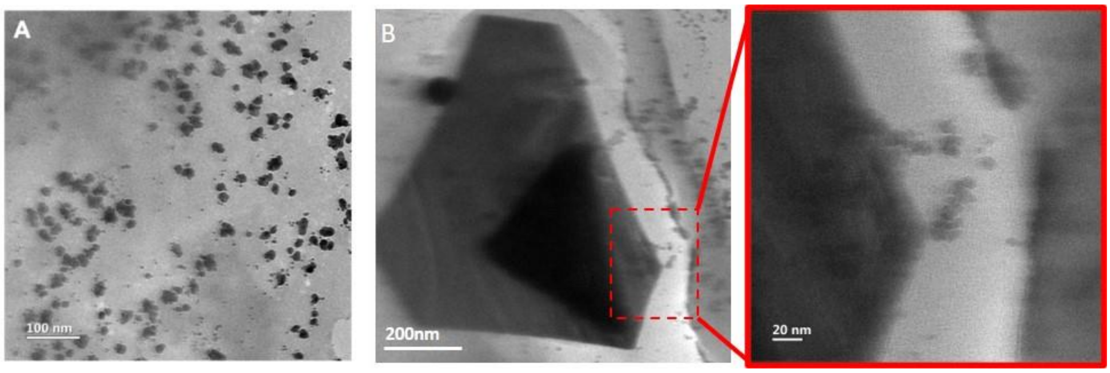

Figure S2. TEM images of reaction kinetic phases of the AuMu NP biosynthesis under pH 3 environment. A) The synthsis starts (phase one) by the formation of spherical particles under k_1 reaction rate. B) The fusion of spherical particles into complex triangular particles under k_2 reaction rate in phase two.

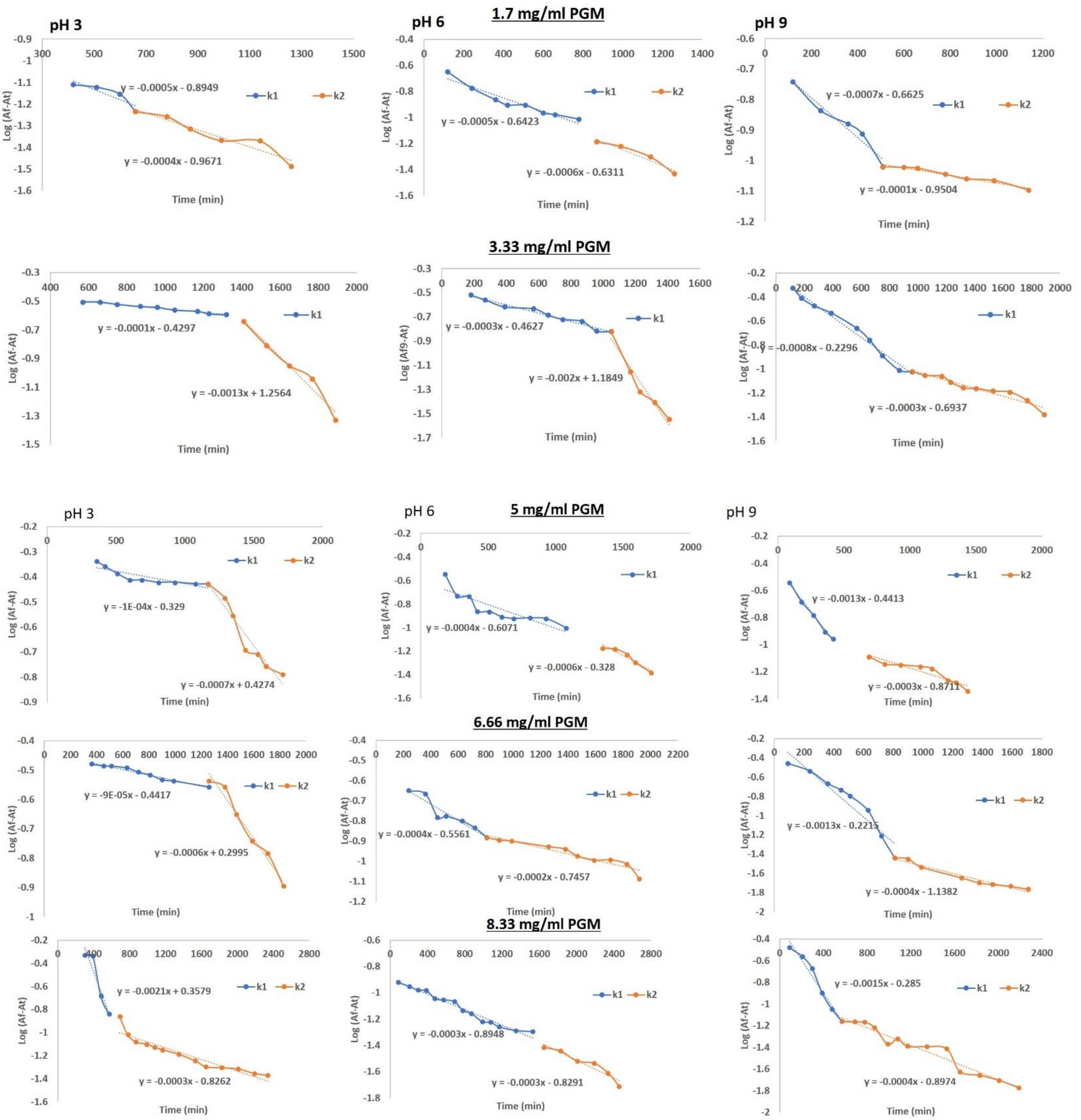

Figure S3. Kinetic analysis from the UV-Vis data obtained from synthesis reaction at different PGM concentrations at different reaction times.

The rate constant k was obtained from the slope of graph of logarithm of maximum AuNP plasmonic absorption peak at the final stage of synthesis reaction minus

maximum AuMu plasmonic absorption peak at the beginning of the reaction in front of the reaction time [5, 45, 46]. The indication of time when the reaction began is the transition of the color from the Au ions + PGM solution: from pale yellow to pink (pH 3), bluish (pH 6) or red (pH 9).

Photothermal properties of the AuMu formulations

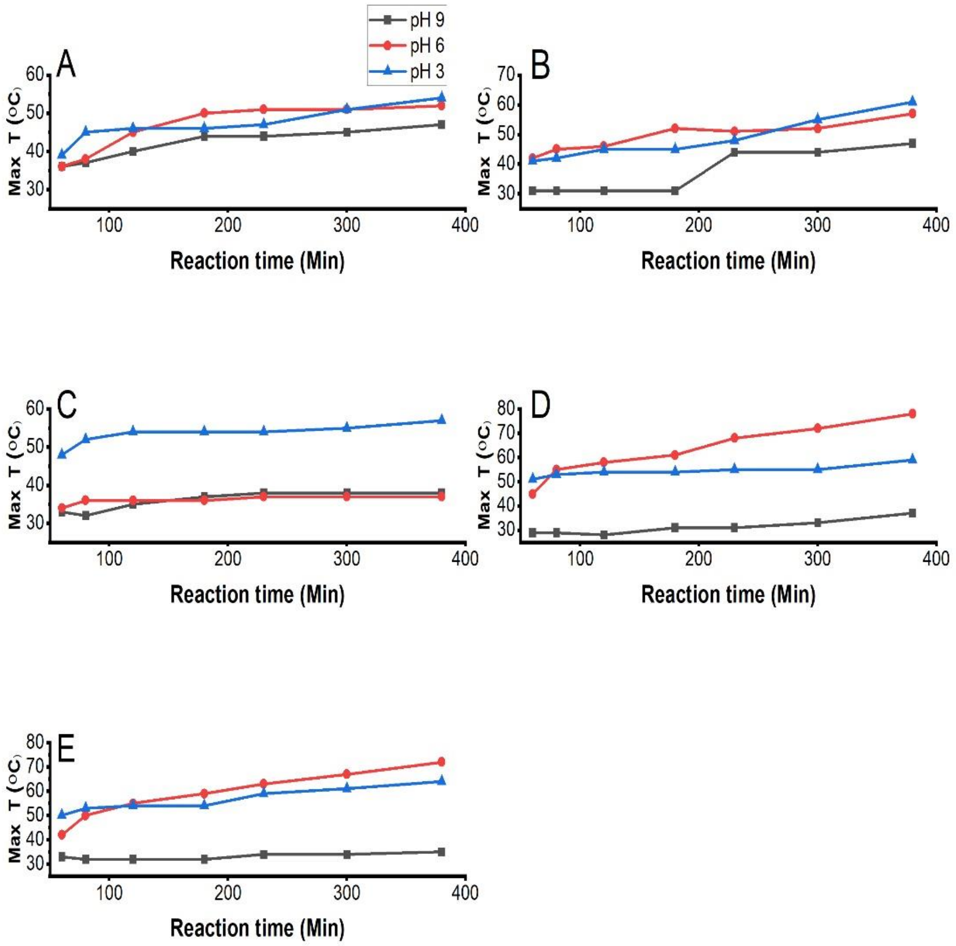

Figure S4. Photothermal properties of the AuMu complexes biosynthesized at different PGM concentration and pH. A) 1.7 mg/ml PGM, B) 3.3 mg/ml PGM, C) 5 mg/ml PGM, D) 6.7 mg/ml PGM, E) 8.3mg/ml PGM. The data show the maximum temperature reached under NIR laser irradiation *vs.* time of the reaction (error of ±1 ºC and ±1 min).

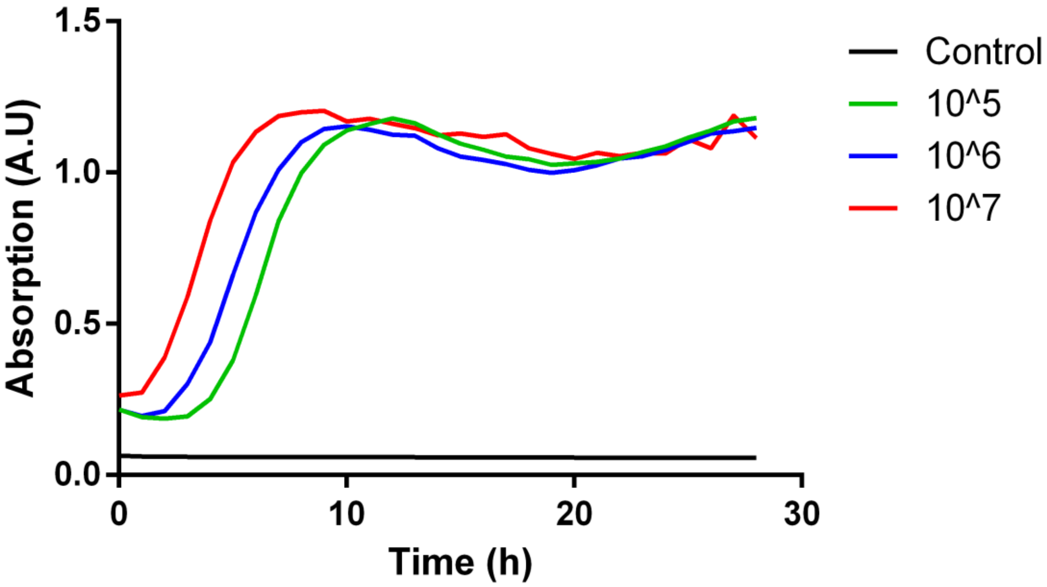

Figure S5. MRSA growth in TS-broth over time in the presence of 1 mg/mL of pure mucin. The MRSA inoculations were prepared at different starting concentrations (10^5, 10^6, and 10^7) with control representing a sterile solution.

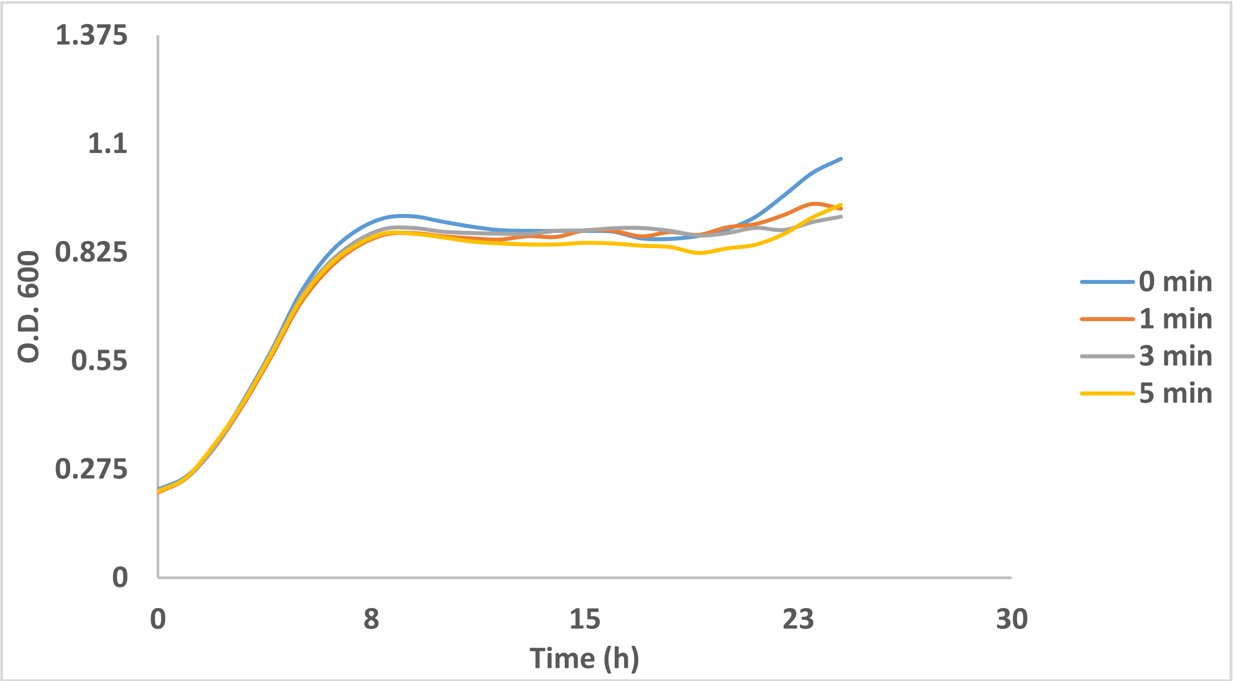

Figure S6. MRSA growth vs. 808 nm laser irradiation for 0, 1, 3, and 5 min in the presence of 6x10-12 M AuNPs (70 nm, biotin terminated)
